# The accessibility of the HSV genome during productive infection can vary in different cell types and affect the outcome of infection

**DOI:** 10.1128/mbio.02987-25

**Published:** 2026-02-13

**Authors:** Jenna M. Nosek, Sarah E. Dremel, Neal A. DeLuca

**Affiliations:** 1 Department of Microbiology and Molecular Genetics, University of Pittsburgh School of Medicine 12317 , Pittsburgh, Pennsylvania, USA; 2 Department of Microbiology, Immunology & Cancer Biology, University of Virginia School of Medicine 12349, Charlottesville, Virginia, USA; Duke University School of Medicine, Durham, North Carolina, USA

**Keywords:** HSV, transcription, ATAC sequencing, accessibility

## Abstract

During productive herpes simplex virus type 1 (HSV-1) infection, the viral genome encounters a number of cellular and viral proteins, such as ICP4, which engage in processes that either attenuate or activate transcription. DNA replication allows the recruitment of cellular transcription factors to late gene promoters, leading to the activation of late gene transcription. We hypothesize that part of the mechanisms by which these viral processes and proteins affect transcription involves altering the accessibility of the genome to the cellular transcription machinery. Using assay for transposase accessible chromatin sequencing (ATAC-seq) to measure accessibility in diploid human lung fibroblasts (MRC5 cells), we found that ICP4 function decreased the accessibility of immediate early genes and increased the accessibility of early and late genes, consistent with the expression of these genes. We also measured accessibility before and after the onset of genome replication. Our results show that the viral genomes were most accessible just after the onset of replication. We conducted similar experiments in a human sensory neuron model (HD10.6) and found that viral genomes were most accessible immediately following infection, and accessibility was reduced as infection proceeded. This finding correlated with viral gene expression and infection outcome, which were considerably reduced compared with that seen in MRC5 cells. ATAC-seq fragment patterns were not consistent with a canonical nucleosome structure for the viral genome, in either cell type, pre- or post-replication. We conclude that accessibility is an important determinant of HSV-1 transcriptional activity and infection outcome.

**IMPORTANCE:**

The transcription of herpes simplex virus type 1 (HSV-1) genes is regulated by viral and cellular transcription factors and genome replication. One regulatory aspect is accessibility of viral genes to the host transcription machinery. In this study, we determine how the major HSV-1 transcriptional regulatory protein, ICP4, and viral DNA replication affect accessibility, and how this relates to viral gene transcription. We also assessed viral genome accessibility in a sensory neuronal model that has the potential for viral gene silencing and establishment of quiescent or latent infection. We conclude that the accessibility of the viral genome to the cellular machinery responsible for viral gene expression is an important determinant to infection outcome.

## INTRODUCTION

 Herpes simplex virus type 1 (HSV-1) is a double-stranded DNA virus with a linear genome of approximately 152 kb (1–3) that replicates in the nucleus of infected cells. The virus subverts cellular pathways to facilitate efficient viral transcription using the RNA polymerase II (RNA polII) transcription machinery of the cell (4). This prolific, productive infection mode is contrasted by a relatively silent (latent) infection in sensory neurons, from which the virus can periodically reactivate.

During the productive phase of infection, viral gene expression is temporally regulated. The genes of HSV-1 can be categorized roughly into three temporal gene classes that are transcribed in a regulated cascade (5, 6). Before *de novo* protein synthesis, immediate early (IE) genes are efficiently transcribed due to the binding of VP16 in the promoter regions of IE genes (7–9). The accumulating IE gene products are required to activate early (E) gene transcription (6). E gene products include the replication machinery needed to synthesize new viral genomes (10, 11). Viral genome replication and IE proteins are required for late (L) gene transcription (6, 12, 13). L proteins comprise the virion structure and proteins needed for assembly. L genes can be further classified into leaky L or true L gene products depending on how tightly their expression is tied to viral DNA replication (13, 14).

While the transcription of cellular and latent HSV-1 genomes is constrained by classical chromatin (15, 16), the structure of HSV-1 genomes in the productive state is less clear. Some studies conclude nucleosome or nucleosome-like structures are present on viral genomes during productive infection, particularly early in infection (15, 17–20). Other studies find either relatively low histone occupancy and/or little evidence for nucleosomal structure in productive infection, including at 1 and 2 h post-infection (hpi) (21–24). Therefore, while the accessibility of the cellular genome is controlled by classical chromatin, how genome accessibility is controlled during productive infection is unclear.

Many proteins, both cellular and viral, interact with the HSV-1 genome throughout productive infection (24). At 1 hpi in diploid human fibroblasts, IE genes begin to be transcribed from the viral genome. The genome is also found in association with interferon-γ inducible factor 16 (IFI16) and promyelocytic leukemia (PML) nuclear bodies (NBs), including α-thalassemia X-linked intellectual disability (ATRX), which function to silence viral transcription (25, 26). By 2 hpi, infected cell polypeptide 0 (ICP0) has targeted host defense proteins, including PML NBs for degradation (27–30). Another critical IE protein necessary for robust E and L gene transcription is ICP4 (31). ICP4 is required for continuous, robust transcription of early and late genes, as well as infectivity (21, 32–34). ICP4 readily binds to the viral genome (21, 35, 36) and recruits RNA polII transcription factors, including the mediator complex and transcription factor II D (TFIID) (24, 37, 38). However, it can also repress transcription depending upon where it binds relative to a gene promoter (36, 39–41). Importantly, early in infection, ICP4 densely binds to the viral genome due to its high abundance relative to viral DNA copy number (21) and its ability to multimerize on DNA (42). As the genome replicates, its binding density decreases such that it only is found bound to the highest affinity binding sites on the viral genome (21). How ICP4 affects the accessibility of the viral genome to other proteins requires further study.

Upon sufficient expression of E genes, the viral replication machinery promotes the onset of genome replication. PolII transcription initiation complexes bind to promoters of late genes as a function of DNA replication (12). The replication of the viral genome and ICP4 are crucial for the shift towards late gene transcription. The onset of viral genome replication causes a dramatic change in the transcriptional landscape of HSV-1 (43). How replication affects the accessibility of the viral genome to the transcription machinery requires further study.

We hypothesize that viral processes and/or proteins affect the accessibility of the genome to the cellular transcription machinery contributing to the regulated cascade of HSV transcription. In this study, we utilized assay for transposase accessible chromatin sequencing (ATAC-seq; assay for transposase accessible chromatin) (44) to determine accessibility of HSV-1 genome during productive infection. We evaluated how ICP4 and viral processes, such as replication and packaging, may affect genome accessibility. Additionally, we determined the accessibility of the genome during infection of a neuronal cell model to determine how gene expression and infection outcome may differ in a different physiological cellular context.

## RESULTS

** **The purpose of this study was to determine the accessibility of the HSV-1 genome during productive infection and correlate this information with the known cascade of transcription. One landmark with respect to transitions in this cascade is viral DNA replication, which ushers in the transition from early to late gene expression (13). It has been shown that the onset of DNA replication is sufficient to license late gene expression (43) and allow the cellular transcription machinery to bind to mRNA promoters (12). To guide our accessibility studies, we first assessed the onset of viral replication during productive HSV-1 infection.

**Fig 1:**
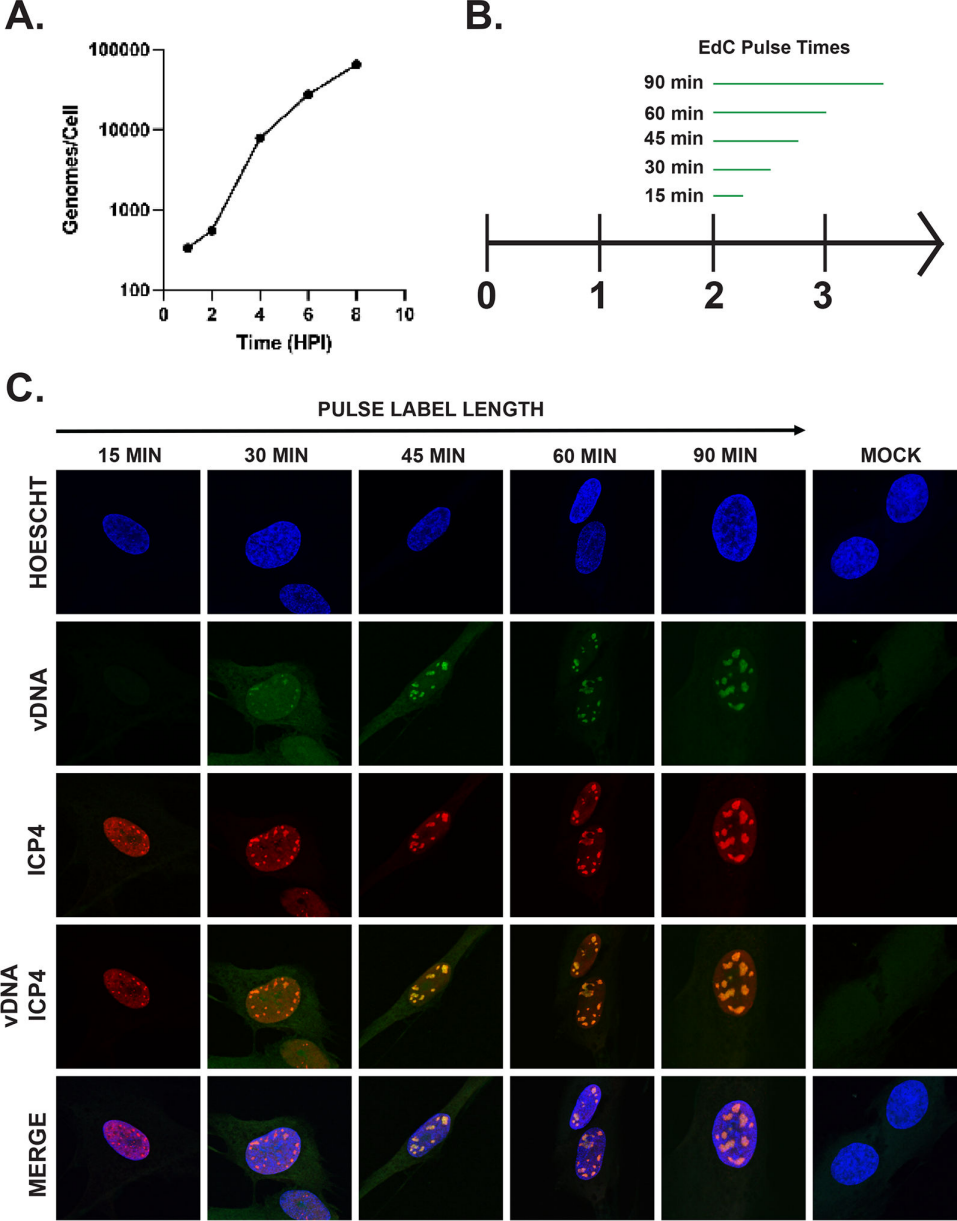
Identifying the onset of viral genome replication. (**A**) Quantification of viral genomes measured by qPCR as a function of time post-infection. (**B**) Schematic of pulse labeling of MRC5 cells during a productive infection. Cells were infected with KOS, and at 2 hpi, cells were pulse-labeled with EdC for 15 to 90 min. Cells were then harvested and processed for confocal microscopy. (**C**) MRC5 cells infected with KOS were pulsed with EdC starting at 2 hpi for various lengths of time. Cellular DNA was visualized by Hoechst staining, replicated viral genomes (vDNA) by click chemistry with EdC, and ICP4 by immunofluorescence. The merge panel demonstrates the localization of viral DNA and ICP4 with respect to cellular DNA.

We performed quantitative PCR (qPCR) on isolated nuclei sampled throughout the first 12 h of a productive infection (Fig. 1A). Viral genomes increased rapidly from 2 to 4 hpi, indicating the start of HSV-1 replication. After 4 hpi, the quantity of viral genomes increased steadily as a result of ongoing viral replication. The labeling of viral genomes with ethynyl-modified nucleosides has been used to identify and track nascent viral genomes in infected cells (45, 46). To further investigate when DNA replication starts in our system, we designed a time course to view the emergence of nascent viral genomes in replication compartments, as first described by de Bruyn Kops and Knipe (47). MRC5 cells were infected with HSV-1 strain KOS for 2 h (Fig. 1B). At 2 h, the cultures were labeled with EdC for various increments of time ranging from 15 to 90 min. Figure 1C shows EdC-labeled genomes relative to ICP4 expression. ICP4 was visualized as small discrete foci in the 15-min pulse. This is consistent with ICP4 being expressed by 2 hpi and binding to input genomes. Nascent DNA was not detected at this time. Distinct foci of nascent DNA emerged at 2.5 h (30-min pulse) in the nuclei of infected cells. These foci contained labeled viral DNA (green) that consistently colocalized with ICP4 (red). As EdC pulse times increased, the replication compartments continued to increase in size. These data illustrate that viral replication began as early as 2.5 h and provided us with a detailed timeline for the onset of viral genome replication.

** ** To determine the accessibility of viral genomes, we utilized ATAC-seq (44). The transposase (TN5) cuts and inserts sequencing primers at positions in the viral and cellular genomes that are accessible. Accessibility can be restricted by chromatin or other DNA-binding proteins. Therefore, ATAC-seq can be utilized to investigate open chromatin regions, nucleosome presence, and DNA-binding proteins on the genome, making it an ideal tool to employ in these studies (44).

Productive infection by HSV-1 in many cell types, such as MRC5 is relatively rapid. Distinct, but overlapping sets of viral and cellular proteins function on the infecting genome at different times post infection (24), and nascent virions can be detected by 6 hpi. Most ATAC-seq protocols involve the treatment of isolated nuclei with TN5. The rapid and dynamic nature of infection might complicate interpretations of standard ATAC-seq experiments. Therefore, we entertained the use of crosslinking prior to nuclei isolation. We hypothesized that ATAC-seq on nuclei isolated from crosslinked samples would enable observation of more transient protein-DNA interactions that may affect accessibility. Therefore, we performed a pilot experiment comparing the results of ATAC-seq with and without crosslinking.

**Fig 2:**
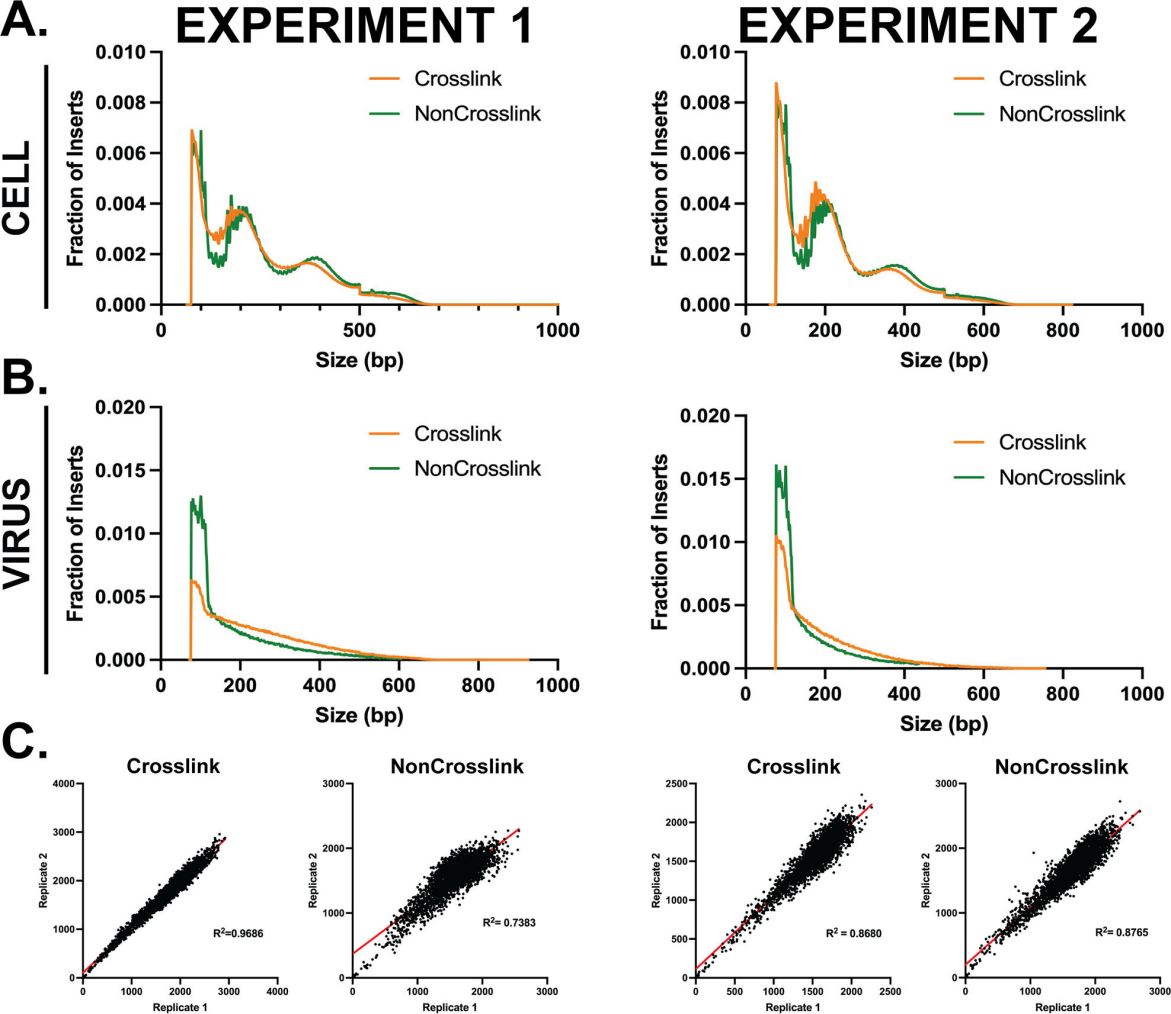
Comparison of non-crosslinked and crosslinked ATAC-seq. MRC5 cells were infected with KOS and harvested at 3 hpi with or without formaldehyde crosslinking and subject to ATAC-seq. Shown are the results of two experiments, with infections performed in duplicate for each experiment. For parts A and B, the average of the duplicates is shown. (**A**) Histogram plot of ATAC-Seq insert fragment size for crosslinked (orange) and non-crosslinked (green) reads mapped to the cellular genome. (**B**) Histogram plot of ATAC-seq insert fragment size for crosslinked (orange) and mock-crosslinked (green) reads mapped to the viral genome. (**C**) Reproducibility plot comparing the duplicate samples for crosslinked and non-crosslinked internal duplicates with R squared values.

MRC5 cells were infected with HSV-1 (strain KOS) for 3 h. One set of cultures was crosslinked with formaldehyde, and another was not crosslinked. Nuclei were isolated and reacted with TN5, and the samples were further processed as described in Materials and Methods. Fig. 2 shows the results of two independent experiments each performed in duplicate. For both experiments, a repeating pattern indicative of nucleosome structures was evident for reads mapped to the cellular genome (Fig. 2A). This corresponds to peaks roughly occurring at 190–200 and 360–400 bp, which is indicative of mono- and di-nucleosomes, respectively. A fine, repetitive structure was also evident (Fig. 2A; Fig. S1A), indicative of some degree of cutting by TN5 on one side of the nucleosomes. When comparing the size of fragments mapped to the cellular or viral genome, it is clear that viral genomes lack the same oscillating pattern. Viral genomes exhibited a monotonic decrease in fraction of inserts with a given insert size. Non-crosslinked viral genomes gave rise to more short inserts, indicating increased TN5 cutting and genomic accessibility as compared with crosslinked samples (Fig. 2B). Crosslinked samples had increased spread for viral genome fragment distribution. Additionally, when comparing overall genome accessibility across the HSV-1 genome, the crosslinked and non-crosslinked samples were very similar, with the crosslinked samples exhibiting more fine structure and the mock-crosslinked samples more uniform. (Fig. S1B). When comparing the reproducibility of these samples, crosslinked replicate samples were more consistent than non-crosslinked (Fig. 2C). These data indicate that the viral genome appears more accessible when it is not crosslinked, possibly due to the transient nature of the genome interactions in the more dynamic viral system. Crosslinking may preserve transient interactions taking place that in turn are able to affect the accessibility of the viral genome. Furthermore, replicates are more reproducible with crosslinked samples. Therefore, we used the crosslinking protocol in all future experiments to promote capture of transient interactions with the viral genomes.

### Accessibility of the viral genome as a function of ICP4

**Fig 3:**
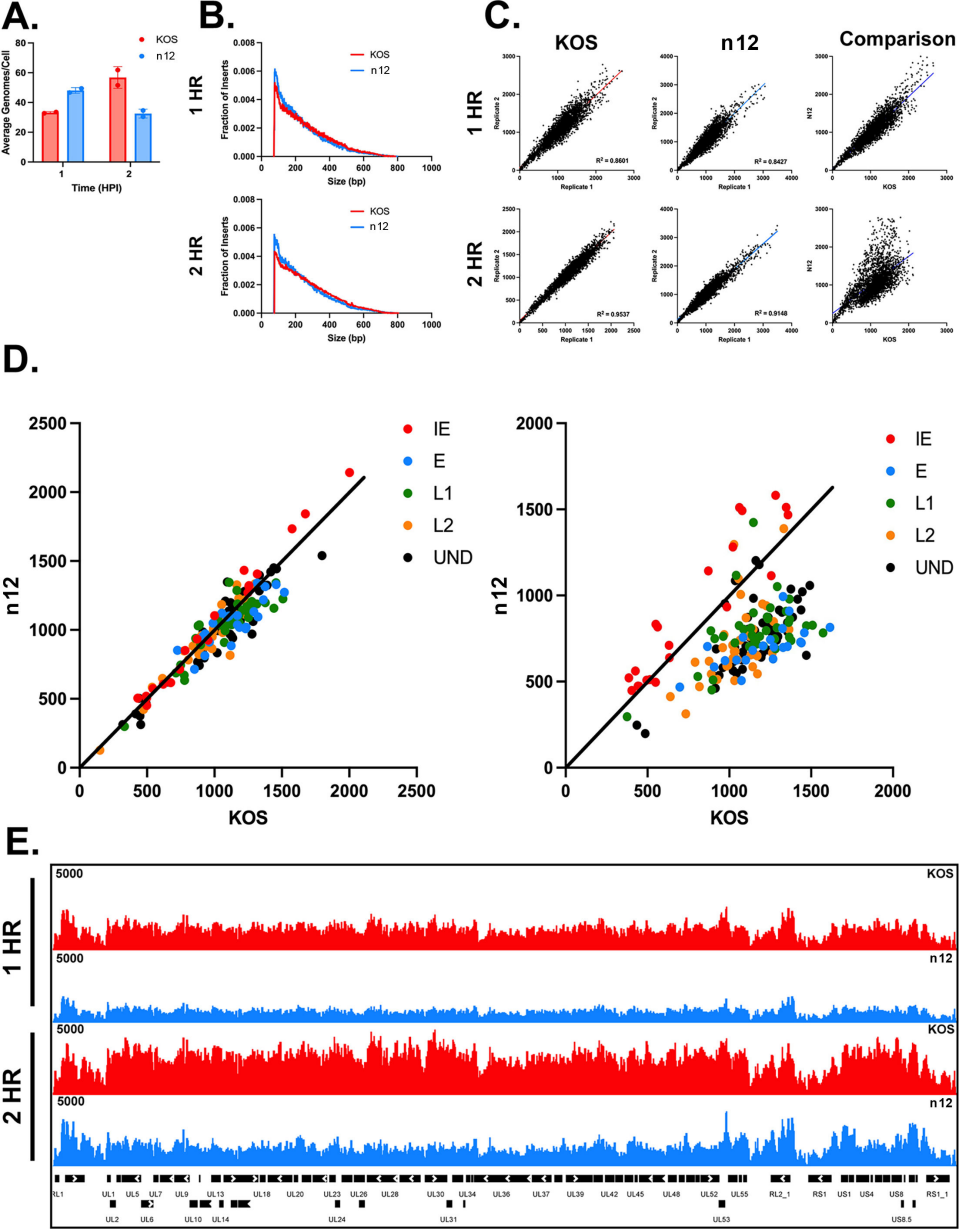
Effect of ICP4 on the accessibility of the HSV genome. MRC5 cells were infected with KOS (WT) and n12 (ICP4 mutant). Cells were harvested at 1 and 2 hpi and processed for ATAC-seq. Infections were performed in duplicate and separately analyzed by ATAC-seq. For panels **B**, **D**, and **E**, the average of the duplicates is shown. (**A**) Quantification of KOS and n12 viral genomes by qPCR. (**B**) Histogram plot of the fraction of insert fragment size aligned to the KOS viral genome for KOS and n12. (**C**) Reproducibility plots comparing replicates for KOS and n12 at 1 and 2 hpi with a bin size of 50 normalized to sequencing depth and viral reads. Comparison of averaged KOS and n12 bigwigs. (**D**) Plots comparing averaged KOS and n12 bigwigs sorted by temporal gene glass at 1 and 2 hpi with a bin size of 1,000. (**E**) ATAC-seq bigwig alignment to the KOS viral genome normalized to sequencing depth and viral reads per cell comparing KOS and n12 at 1 and 2 hpi.

Given that ICP4 is essential for efficient transcription of E and L genes, and that it binds the viral genome during productive infection (21), we wanted to determine if and how ICP4 affects genome accessibility early in infection. We compared the ability of ICP4 to affect accessibility by performing ATAC-seq in duplicate on cells infected with wild-type virus (KOS) and an ICP4 mutant (n12), at 1 and 2 hpi. We did not go beyond 2 hpi because the genome of WT virus would replicate and that of n12 would not. qPCR was performed on the same nuclei as the ATAC-seq to determine the quantity of viral genomes per nucleus (Fig. 3A). The insert size distribution for n12 and KOS was similar, with a slight shift toward smaller insert sizes for n12 (Fig. 3B).

Comparison of the insert size distribution in the duplicate samples demonstrated the reproducibility of the ATAC-seq samples in this experiment (Fig. 3C). However, when comparing the averaged replicates for KOS to n12, while they were similar at one hpi, multiple populations emerged at 2 hpi (Fig. 3C). Upon changing the bin size and sorting the bins by temporal gene class, we observed that the population that was more accessible in n12 is comprised primarily of IE genes at 2 hpi (Fig. 3D). The population that was more accessible in KOS than in n12 was comprised mainly of E and L genes. When looking at the accessibility across the genome at one hpi, there was little difference in the pattern; however, it appears that KOS was slightly more accessible (Fig. 3E). At 2 hpi, KOS reads were more evenly distributed across the viral genome and more accessible when compared with n12 reads. In n12 at 2 hpi, the overall accessibility across the viral genome showed peaks. These peaks indicate more accessible regions in n12 and overlap with immediate early genes, specifically ICP0, ICP22, ICP27, and ICP47 (Fig. 3D). Therefore, the presence of ICP4 affects accessibility in a manner consistent with its role in the expression of IE and later genes.

### Accessibility of the viral genome as a function of viral genome replication

**Fig 4:**
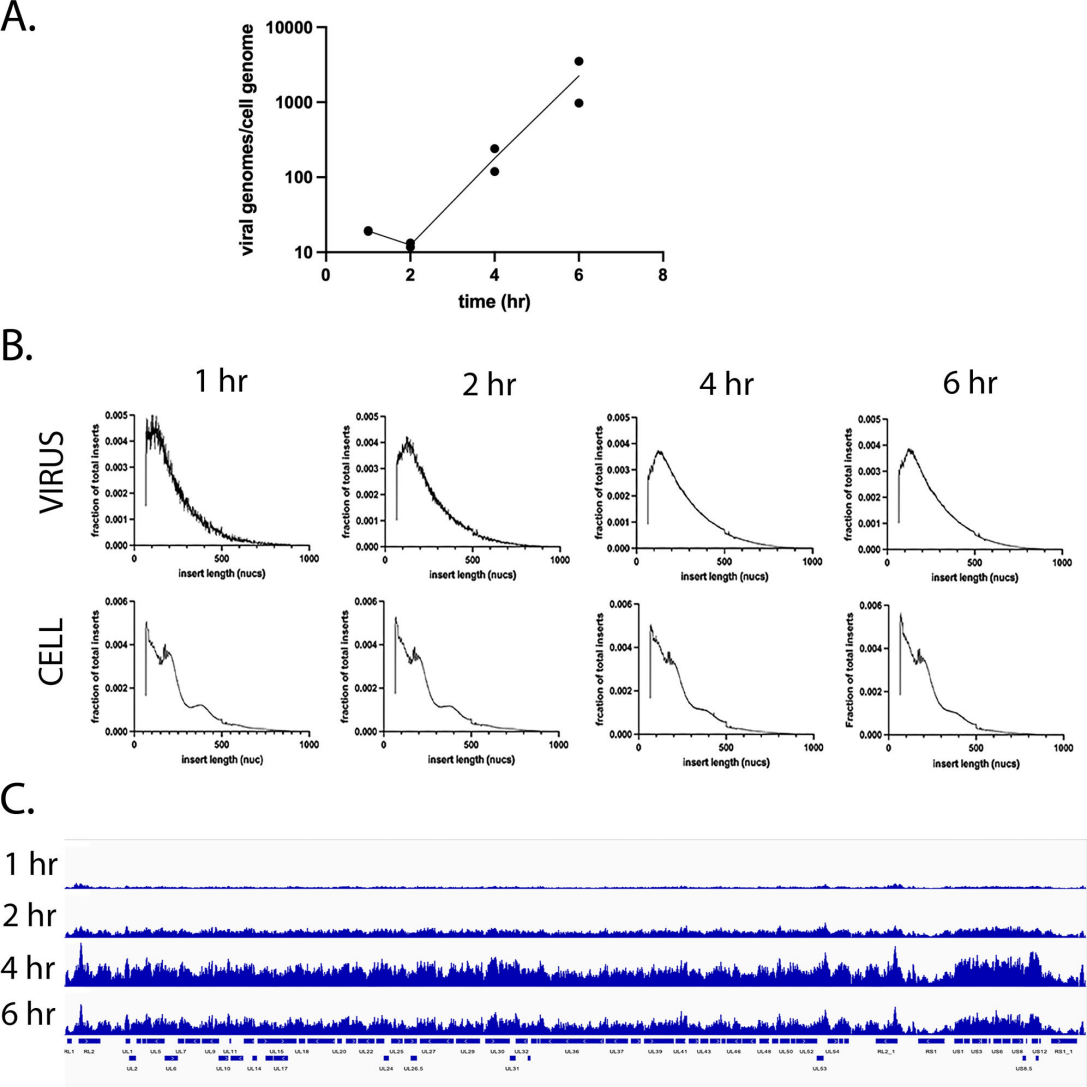
Comparison of genome accessibility before and after the onset of genome replication. MRC5 cells were infected with KOS for 1, 2, 4, and 6 hpi and processed for ATAC-seq. Infections were performed in duplicate and separately analyzed by ATAC-seq. For panels B and C, the average of the duplicates is shown. (**A**) qPCR quantification of the number of genomes per cell nucleus. (**B**) Plots of the fraction of inserts having a given length for the viral and cellular genomes in the same samples. (**C**) ATAC-Seq bigwig alignment to the KOS viral genome normalized to viral reads per cell and cell reads. The maximum for all four plots is 29,000 reads.

ICP4 activates the transcription of viral early genes, including those involved in the replication of the HSV-1 genome (10). Replication allows for the binding of the cellular transcription machinery to viral late gene promoters (12). We hypothesized that replication may increase the accessibility of the genome allowing transcription factors greater access. To determine how replication affects accessibility, we performed ATAC-seq and quantitative PCR at 1, 2, 4, and 6 hpi, in duplicate. These times represent accessibility prior to replication (1 and 2 h), shortly after the onset of replication (4 h), and during ongoing replication, and subsequent steps in the viral production cycle, including packaging (6 h). At 1 and 2 hpi, the number of genomes per nucleus remained relatively constant (Fig. 4A). Between 2 and 4 hpi, the number of viral genomes per nucleus increased rapidly, indicative of genome replication. This trend continued at 6 hpi. The ATAC-seq replicates were fairly reproducible (Fig. S2A). The scatter in the 1 hpi sample is due to the relatively low number of mapped viral reads.

The observed insert size distribution was consistent with the presence of nucleosomes on the cellular genomes (Fig. 4B). However, the observed pattern on the viral genome was not consistent with the presence of regular nucleosomes at any of the time points. Normalizing the traces to viral reads and plotting them across the genome (Fig. S2) revealed more accessibility at 1 hpi on IE genes consistent with the findings in Fig. 3E. Tagmentation was more spread out and less focused on IE genes at later times. However, when the ATAC-seq reads were normalized to the number of genomes per nucleus (Fig. 4A), an interesting pattern emerged (Fig. 4C). The accessibility of the genome at 1 and 2 hpi was relatively low, with 1 hpi being less accessible than 2 hpi. However, at 4 hpi, despite a 10-fold increase in the numbers of viral genomes, the accessibility of these genomes greatly increased. This was also observed at 6 hpi, with a small decrease in accessibility relative to the 4 hpi samples. This may be due to the influence of genome packaging and/or the presence of a different population of less accessible genomes.

**Fig 5:**
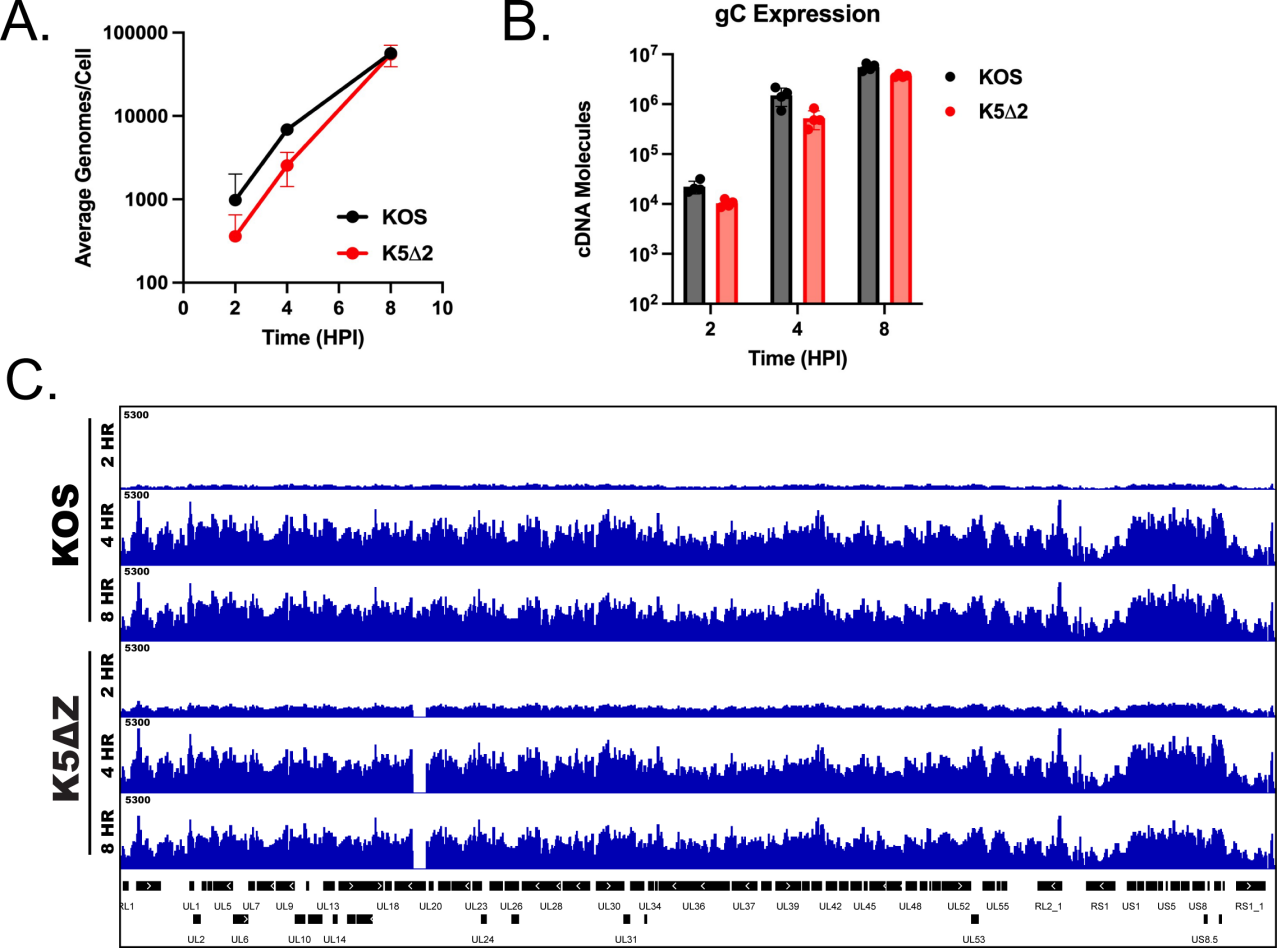
Effect of packaging on ATAC-seq measurements. MRC5 cells were infected with KOS and K5ΔZ and harvested at 2, 4, and 8 hpi and analyzed by ATAC-seq. Infections were performed in duplicate and separately analyzed by ATAC-seq. For panel C, the average of the duplicates is shown. (**A**) Quantification of KOS and K5ΔZ viral genomes by qPCR. (**B**) Expression of a true late gene, gC. RT-qPCR was performed on KOS and K5ΔZ infected cells at 2, 4, and 8 hpi. gC mRNA values were normalized to GAPDH mRNA values. (**C**) ATAC-seq bigwig alignment to the KOS viral genome for KOS and K5ΔZ normalized to viral genomes per cell and cell reads.

To determine the effect of packaging on the accessibility measurements in Fig. 4, we compared KOS to a capsid mutant, K5ΔZ (48). This virus has a deletion in UL19, which encodes for VP5. VP5 is the major capsid protein for HSV-1. When VP5 is not present, the capsid cannot form, and thus, newly synthesized genomes cannot be packaged. We performed qPCR to assess genome replication and mRNA accumulation in the mutant, respectively. Consistent with previous studies (48), K5ΔZ is genome replication-competent (Fig. 5A). In addition, late gene expression was similar to KOS, as measured by gC mRNA accumulation (Fig. 5B). An ATAC-seq experiment was performed to measure the accessibility of KOS compared with the viral capsid mutant, K5ΔZ, at 2, 4, and 8 hpi. As seen previously, viral replication occurs between 2 to 4 h and continues thereafter (8 hpi). Genome accessibility at the three time points was measured and normalized to viral genomes per cell. At 2 h, accessibility was low for both viral strains (Fig. 5C). Like in Fig. 4C, viral genomes were most accessible regardless of viral strain at 4 hpi. At 8 h, accessibility was similar for KOS and K5ΔZ. We conclude that viral packaging is not a significant contributor to genome accessibility at these times post-infection.

### Accessibility of viral genomes in a sensory neuron model

**Fig 6:**
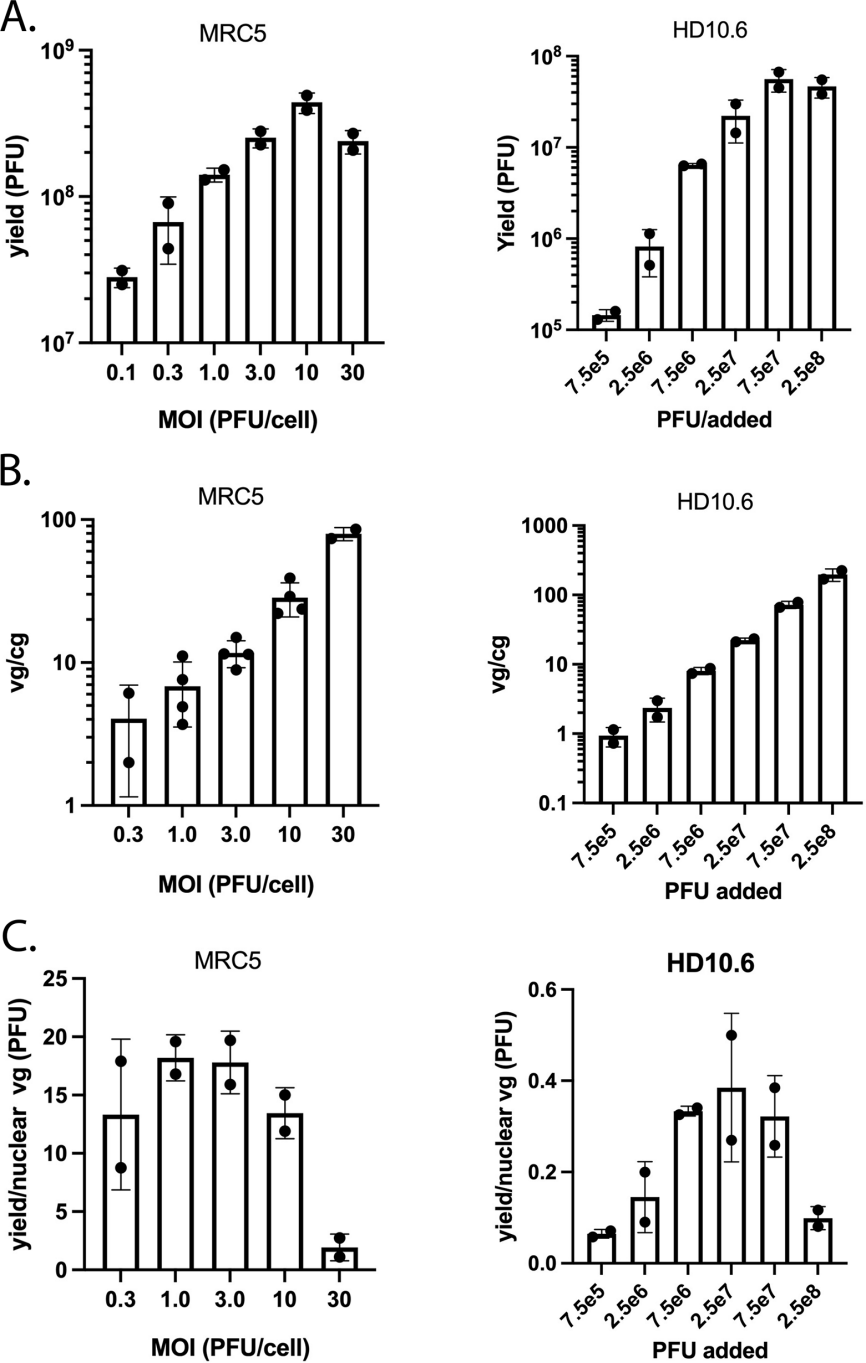
Comparison of infection parameters for MRC5 cells and differentiated HD10.6 cells. (**A and B**) KOS was used to infect 35-mm dishes of MRC5 cells or differentiated HD10.6 cells in duplicate with the indicated amounts of virus. (**A**) The infected cells were harvested at 24 hpi, and virus yield was determined by plaque assay on Vero cells. (**B**) The infected cells were harvested at 2 hpi, and nuclei were prepared from which total DNA was prepared and quantified for cellular and viral genomes by qPCR. (**C**) The viral yield at 24 hpi per nuclear genome at 2 hpi was calculated from panels A and B.

We next sought to determine the accessibility of the viral genome during productive infection in a model neuronal cell type, reasoning that it may differ from that seen in MRC5 cells and may have some bearing on the outcome of infection or the eventual establishment of latency in sensory neurons. Thellman et al. (49) have characterized an immortalized human dorsal root ganglion cell line (HD10.6) with respect to HSV-1 latency and reactivation. They found that the differentiated cells express neuron-associated markers, supported delayed productive infection, and that HSV-1 can establish latency and reactivate from them. We were interested in productive infection subsequent to viral entry and through the onset of genome replication. The parameters of productive infection in HD10.6 cells are not well established. Therefore, we first had to establish the mechanics of infection of differentiated HD10.6 cells. The nerve cell bodies are smaller than MRC5 cells, and much of the monolayer is comprised of axons and empty space (see Fig. S3A). Furthermore, the attachment of the cell monolayer to the coated surface is not as strong as MRC5 cells, making it sensitive to shearing forces during the attachment period. To minimize the effects of shear forces, the adsorption volume for the differentiated HD10.6 cells was 2.5 times that used for MRC5 cells. Therefore, while it is straightforward to ascribe a multiplicity of infection (MOI) for MRC5 cells, virus input on differentiated HD10.6 cells is more operationally defined. Accordingly, we compared the yield of virus after 24 hpi and the number of genomes per cell nucleus after 2 hpi of infection of MRC5 and differentiated HD10.6 cells as a function of input virus (Fig. 6). There were about 1 × 10^5^ and 5 × 10^4^ cells/cm^2^ for MRC5 and HD10.6 cell monolayers, respectively. The amount of virus used for the infections depicted in Fig. 6 is given as MOI (PFU/cell) and absolute virus numbers (PFU) for MRC5 and HD10.6 cells, respectively.

 For both cell models, the total yield of virus increased with increasing input until it eventually plateaued at the highest inputs (Fig. 6A). Despite adding considerably more virus to the HD10.6 than to the MRC5 monolayers, the yields of virus produced in MRC5 cells were considerably greater. The same experiment was conducted to determine the number of viral genomes per cell nucleus (represented as viral genomes/cell genome) as a function of virus input (Fig. 6B). Again, for both MRC5 and HD10.6 cell,s the numbers of viral genomes per nucleus increased with increasing input; however, it did not level off at the high input. Additionally, unlike the yield experiment (Fig. 6A), the numbers of viral genomes per nucleus at 2 hpi (Fig. 6B) in MRC5 and HD10.6 cells were comparable. Using the data from Fig. 6A and B and plotting it as yield per input nuclear viral genome (at 2 h) result in Fig. 6C. The yield of virus per input viral genome in HD10.6 cells was approximately 40-fold lower than in MRC5 cells. For all subsequent infections of HD10.6 cells, we chose the virus input that gave the greatest yield in HD10.6 cells (9.4 × 10^6^ PFU/cm^2^ of monolayer).

 It is possible that the low yield at 24 h in HD10.6 cells was due to a delay in the replication cycle. However, when virus production was examined over a time course, it was similar from days 1 to 3, with a drop at 4 days (Fig. S3B). It is also possible that not all genomes go on to express viral proteins. When HD10.6 cells were stained for ICP4, most, but not all, cells stained positive for ICP4 at 2 and 6 hpi (Fig. S3C). Interestingly, while the 6 hpi staining resembled replication compartments as one would see in MRC5 cells, the 2 hpi staining was more diffuse than one would see in MRC5 cells. How this may affect the function of ICP4 remains to be determined.

**Fig 7:**
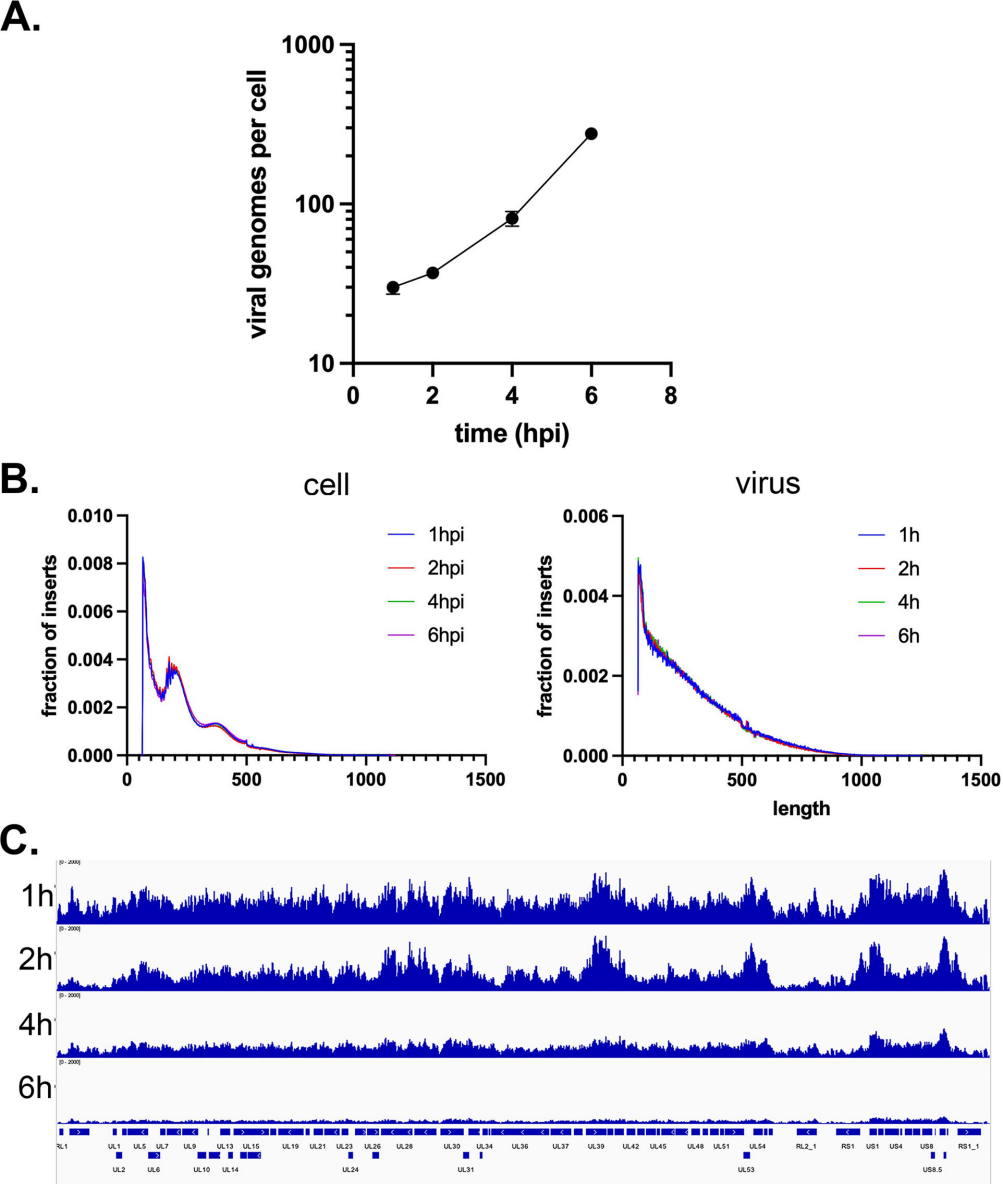
Viral genome accessibility in differentiated HD10.6 cells. Differentiated HD10.6 cells in 60-mm dishes were infected with 1.75 × 10^8^ PFU KOS for 1, 2, 4, and 6 hpi and processed for ATAC-seq. Infections were performed in duplicate and separately analyzed by ATAC-seq. For parts B and C, the average of the duplicates is shown. (**A**) qPCR quantification of the number of genomes per cell nucleus in each of the duplicates. (**B**) Plots of the fraction of inserts having a given length for the viral and cellular genomes for all the time points. (**C**) ATAC-seq bigwig alignment to the KOS viral genome normalized to viral reads per cell and cell reads.

 We went on to determine the accessibility of the HSV-1 genome upon infection of differentiated HD10.6 cells. Duplicate monolayers of infected cells were harvested at 1, 2, 4 and 6 hpi and subjected to ATAC-seq analysis. The number of HSV-1 genomes per cell increased approximately 10-fold over the 6 h of infection (Fig. 7A). This is ~1-fold less robust than replication observed in MRC5-infected cells (Fig. 4A) in the same time period. The duplicate ATAC-seq samples for each time point were very reproducible (Fig. S4A). The fragment size distribution at all time points for the host genome was consistent with nucleosome occupancy, whereas the viral genome was not (Fig. 7B). This echoed our prior data from MRC5 infection (Fig. 4B).

 The tagmentation pattern across the viral genome (Fig. S4B) had visible peaks with regions of high accessibility at certain genomic loci (notably IE promoters and UL39-40) pre-replication. These peaks diffused at later infection time points (Fig. 7C; Fig. S4B). When viral reads were normalized to the number of genomes per cell, the average viral genome accessibility was greatest in early infection (1 hpi) and declined thereafter (Fig. 7C). This is very differentfromn what was seen in MRC5 cell (Fig. 4C), where accessibility per genome increased as infection proceeded.

**Fig 8:**
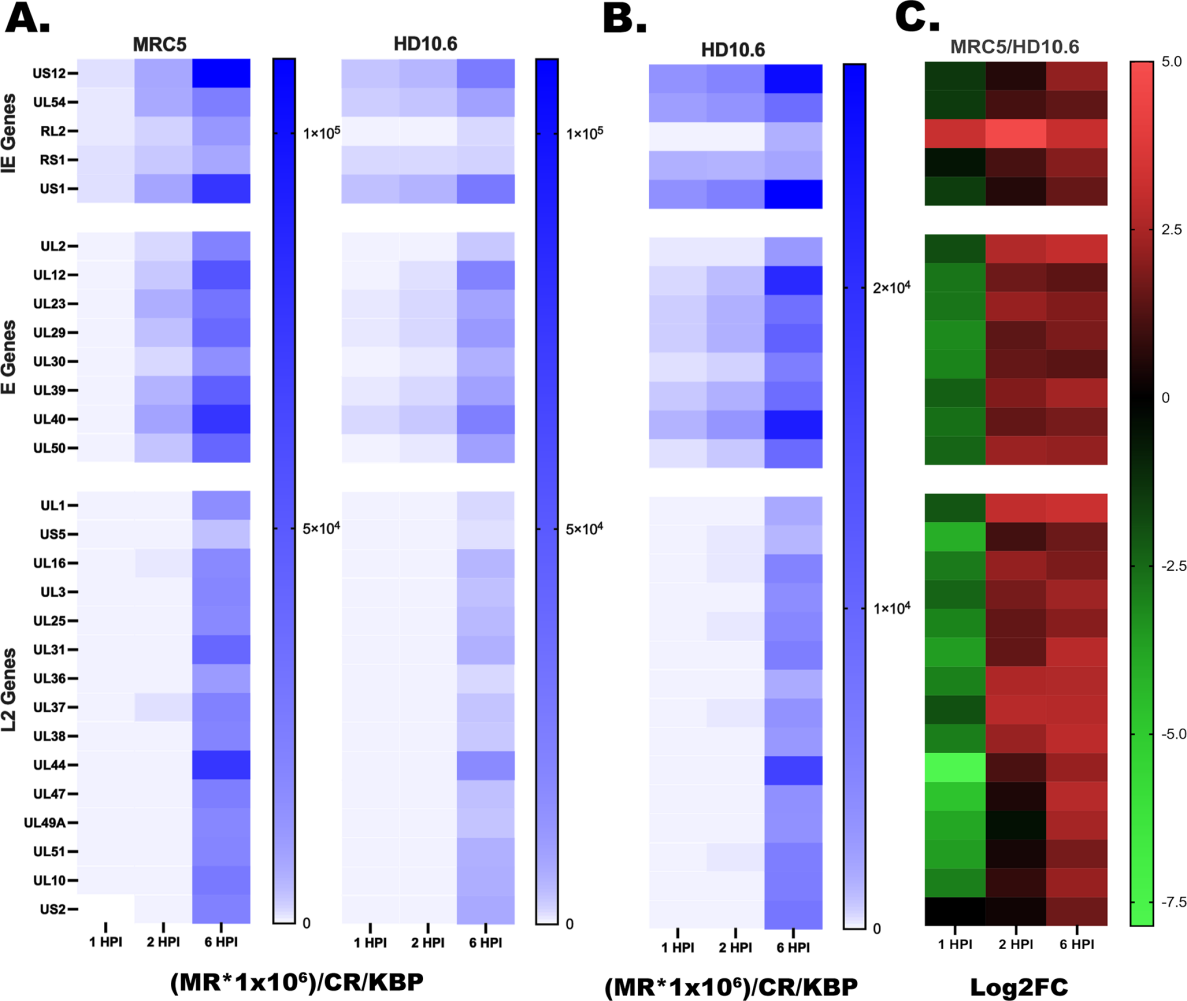
RNA-seq comparison of MRC5 and differentiated HD10.6 cells. Sixty-millimeter dishes of MRC5 cells and differentiated HD10.6 cells were infected at an moi of 10 PFU/cell and 1.75 × 10^8^ PFU of KOS, respectively. At 1, 2, and 6 hpi, total RNA was isolated from the cultures, cDNA prepared, and RNA-seq libraries prepared. Paired-end sequencing was performed, and individual abundance levels were determined for the HSV genes (Table S1). (**A**) Heat maps of expression levels for MRC5 and HD10.6 cells (mapped reads/million reads/kb). (**B**) Part A for HD10.6 cells displayed at a more sensitive scale to more clearly visualize the progression of gene expression. (**C**) Log_2_ fold change of MRC5 over HD10.6 from panel A.

 To determine how the accessibility of the HSV-1 genome may affect gene expression upon infection of differentiated HD10.6 cells, RNA-seq was performed on duplicate cultures infected for 1, ,2 and 6 hpi. MRC5 and differentiated HD10.6 cells were analyzed in parallel. The heatmaps in Fig. 8 depict the results of this experiment for representative IE, E, and L genes (12). Panel A represents a direct comparison of the values for expression in MRC5 and differentiated HD10.6 cells. Panel B is a scale-expanded version of the HD10.6 values depicted in panel A, in order to better observe the progression of gene expression from 1 to 6 hpi in HD10.6 cells. Panel C is a representation of the difference between the expression of the genes in MRC5 versus HD10.6 expressed as the log2 value of MRC5/HD10.6. The values used to create the heatmap in Fig. 8A are given in Table S1. Several conclusions from this data can be drawn: (i) overall the regulatory cascade is similar in HD10.6 cells to that in MRC5 cells, (ii) the expression of individual genes is less in HD10.6 cells than in MRC5 cells at 2 and 6 hpi, and (iii) with the exception of ICP0 (RL2) and ICP4 (RS1), all of the genes are expressed to a greater level in HD10.6 cells at 1 hpi. The significance of these observations with respect to the accessibility of the genomes and the outcome of viral infection is discussed in the next section.

## DISCUSSION

The transcription of eukaryotic genomes is regulated by DNA-binding proteins that either recruit or influence genomic accessibility to the general transcription machinery. For cellular genomes, the predominant modulator of accessibility is classical chromatin structure. Factors that modify or move histones and nucleosomes allow transcription to be regulated in response to the cellular environment. The transcription of DNA virus genomes, such as that of HSV-1, is also tightly regulated. The regulated transcription of the HSV-1 genome during productive infection results in robust production of viral proteins and progeny virions. There are many cell and viral proteins that bind to the HSV-1 genome during infection that could influence the accessibility of the genome to the cellular transcription machinery. In addition, viral genome replication may affect accessibility to allow for late gene transcription (5, 13).

The genes of the different kinetic classes are closely spaced on the HSV-1 genome with no obvious linear distribution. Despite this, transcription is highly regulated and occurs along with replication, making for an extremely dynamic environment where viral and cellular factors are rapidly associating and disassociating with the genome. Many cellular factors are relocated to replication compartments and likely participate in the various stages of the virus life cycle, including replication proteins, transcription factors, epigenetic modifiers, and recombination/repair proteins (24, 43, 46). In these studies, we used ATAC-seq to determine the accessibility of HSV-1 viral genomes during a productive infection, evaluating contributions of the major viral transactivator (ICP4) and genome replication.

### ICP4 alters accessibility

ICP4 is required for activation of viral E and L genes, repression of IE genes, and is one of the most abundant proteins on the genome during productive infection (46). Given its role in transcription and its binding to the viral genome, we determined how ICP4 affects accessibility early in infection and prior to replication.

The relatively high accessibility of IE genes that we observed in the absence of ICP4 is likely attributed to VP16 recruiting chromatin modifiers (18, 50), and other activators of IE genes, which ICP4 antagonizes. The repressive effect of ICP4 was shown to be dominant over the action of activators on IE genes (39, 51). With non-IE genes, accessibility was greater in the presence of ICP4 at 2 hpi only. This was not seen at 1 hpi most likely due to the low levels of ICP4 present in the nucleus at that time. The increase at 2 hpi might reflect the binding of ICP4 to the viral genome, possibly displacing a repressive nucleoprotein structure, or simply being a function of its activation function recruiting the general transcription machinery.

While many studies have documented the presence of histones on the viral genome or inferred the presence of a noncanonical or unstable nucleosome structure (17), and that ICP4 mobilizes histones (52), evidence for classic nucleosomal structure during productive infection has not been documented (21, 23, 24). Herein, our findings argue against the presence of classic nucleosome structures, in the presence and absence of ICP4. However, both VP16 and ICP0 have been shown to influence removal and acquisition of histone marks (50, 52–55) and affect accessibility (56, 57). It is possible that at early times post-infection, histones participate in some form of nucleoprotein along with other viral and cellular proteins, and this does not involve the formation of classic nucleosomes. We conclude that ICP4 function affects the accessibility of IE and non-IE genes, consistent with its role in the repression and activation of those genes, respectively. The mechanism underlying this transition is under further study.

### Viral genomes are most accessible when replicating

All of the events that occur during infection within the nucleus (transcription, genome replication, virion packaging, and assembly) can be observed within six h of infection of MRC5 cells (58, 59). Following the expression of ICP4 and the expression of E genes, the replication of the genome and the activation of L gene transcription begins. This occurs between 2 and 3 hpi in MRC5 cells. To assess how genome accessibility changes, progressing from entry through genome replication, we performed ATAC-seq.

Viral genomes were more accessible at 2 than at 1 hpi. At 1 hpi, viral genomes are associated with ND10, (PML-containing) structures which encapsulate and repress viral genomes (60). Early in infection, viral genomes are also bound by IFI16 (24, 61), which has been shown to restrict accessibility (62). By 2 hpi, the IE protein ICP0 has directed the degradation of PML, ICP4 has been synthesized and bound to the viral genome, and the process of early gene activation has begun. We have previously shown in iPOND studies that there is a dramatic shift in the cellular proteins that bind to the viral genome going from 1 to 2 hpi, switching from proteins that repress transcription to proteins involved in transcription (24). Therefore, the change in the accessibility between 1 and 2 hpi reflects these events and the transcription of the genome. The results discussed above demonstrate that the action of ICP4 is in part responsible for this increase in accessibility.

The onset of DNA replication results in the activation of late promoters, which minimally consist of a TATA box and an initiator element. Early times in DNA replication are represented by 4 hpi in our studies. This was the time when we found the genome to be the most accessible. ChIP analysis has shown that replication allows for the binding of polII factors and polII to late promoters (12). Furthermore, iPOND studies reveal that polII binds to newly replicated DNA (43). The dynamics of DNA replication could clear the genome of bound proteins, thus allowing better access for transcription factors to the relatively weaker true late promoters. Molecules involved in repression prior to DNA replication could be histones, HSV-1 DNA binding proteins, such as ICP8 (63), or even ICP4 itself. ICP4 can act as a transcriptional repressor, and replication reduces the density of ICP4 binding to the genome (21), and in some cases, replication relieves the repressive effect of ICP4 (64, 65).

With ongoing DNA replication and late transcription, some nascent genomes will be packaged into virions. At 6 hpi, while we observed an increase in viral genome copy number, there was a notable decrease in genome-wide accessibility. We reasoned that this could be because some packaging of viral genomes was occurring at this time. When we used a viral mutant in the major capsid protein, VP5, we observed no difference in the accessibility of viral genome when comparing the mutant with KOS at eight hpi. It may be that the amount of packaged DNA at this time is small relative to other populations of genomes, including replicating genomes. Pulse-chase iPOND studies have revealed a population of unpackaged genomes that do not have replication and transcription factors bound (43). It may be that this population is less accessible to the transcriptional machinery. The fate of this population of genomes is unknown. It is possible that these genomes represent molecules that do not participate further in productive infection. In highly permissive cells, such as MRC5 cells, their presence would be masked or of little consequence. However, in cells that are less permissible to productive infection, this population may have greater biological significance.

### Accessibility of the viral genome in a neuronal cell model

We also wanted to assess how accessibility might influence the outcome of infection in a sensory neuron model where the virus is capable of establishing a latent infection. We first set out to characterize productive infection with respect to kinetics and yield of virus as a function of input and compare it to MRC5 cells. We found that the differentiated HD10.6 cells were considerably less permissive for HSV-1 than MRC5 cells (Fig. 6), and that the infection was more protracted, consistent with previous findings (49). Interestingly, while the number of viral genomes able to enter the nucleus increased with increasing input virus, we observed a maximum for virus yield in both MRC5 and HD10.6 cells. This is consistent with the results from Enquist and colleagues who found a limit on the number of infecting viruses that go on to replicate (66). Importantly, the maximum absolute yield at 24 hpi and the yield per nuclear genome at 2 hpi was 10- and 40-fold lower, respectively, for HD10.6 compared with MRC5 cells. While the time of onset of viral genome replication was similar for MRC5 and HD10.6 cells, the absolute amount of replication was lower for HD10.6 cells. Therefore, differentiated HD10.6 cells are less permissive for productive infection than MRC5 cells. It is also possible that a population of viral genomes in differentiated HD10.6 cells is becoming quiescent. More studies are required to further explore the fate of HSV-1 genomes in this cell line.

 ATAC-seq experiments suggest that unlike what is seen in MRC5 cells, many genomes in HD10.6 cells become relatively inaccessible as infection proceeds, even after viral genome replication has ensued. The viral genomes were most accessible a 1 hpi. Furthermore, there were peaks of accessibility at early times pos- infection corresponding to the boundaries of the Us region, UL39/UL40 (ribonucleotide reductase), UL54 (ICP27), and UL29, UL30 (ICP8, polymerase). The significance of these peaks is not clear at present. The expression levels of viral genes in HD10.6 cells were lower than in MRC5 cell; however, a similar progression through the HSV-1 regulatory cascade was observed. Comparing the relative levels of mRNA in MRC5 to HD10.6 cells reveals higher relative levels of almost all mRNAs in HD10.6 cells at 1 hpi, and higher levels then after in MRC5 cells. Notable exceptions are RS1 (ICP4) and RL2 (ICP0). These are either the same or always higher in MRC5 cells. The higher relative level of expression at 1 h in HD10.6 cells is consistent with the higher level of accessibility of the genome at this time. In addition, the accessibility of the ICP4 and ICP0 loci is among the lowest in HD10.6 cells.

 The infection process in HD10.6 cells may be considerably more complex than in MRC5 cells. In MRC5 cells, most input viral genomes up to a point undergo a productive infection, where the increased accessibility of the genome as a function of genome replication results in the prolific expression of viral proteins and progeny production. We propose that in HD10.6 cells, there exist multiple populations of viral genomes, one population that undergoes a productive infection, such as that seen in MRC5 cells, and another population(s) that is rendered less accessible, such that gene expression from these genomes and virion production is attenuated. In MRC5 cells, there is a switch between 1 and 2 hpi that facilitates the onset of efficient viral replication (24); however, that shift might not occur as efficiently in HD10.6 cells, such that relatively few genomes go on to productive infection. This would explain the reduced accumulation of viral mRNAs and diminished production of infectious progeny. Most of the genomes are rendered inaccessible. Possible mechanisms leading to the decreased accessibility include the binding of IF16 to the genome, the sequestration of genomes at ND10 (60), chromatin (15, 16), the action of ICP8 (63), or even suboptimal expression of ICP4 and ICP0. At the studied time points (1–6 hpi), it likely does not involve the formation of nucleosomal structures since unlike what was seen for the cellular (HD10.6) genome, there was no evidence of nucleosomal structure for the viral genome. Finally, while the observations in this study document the reduced accessibility, mRNA accumulation, and virus yield as infection proceeds, whether this leads to a quiescent or latent infection remains to be determined.

 Cellular and viral mechanisms affect the accessibility of the viral genome and per genome replication potential. In this study, we establish that the presence of ICP4 and viral DNA replication are two such viral mechanisms. We also show that accessibility of the genome is managed differently in distinct physiologically relevant cell types, and this may result in altered permissivity or viral infection outcome.

## MATERIALS AND METHODS

### Cells and virus

MRC5 (human fetal lung) and Vero (African green monkey kidney) cells were obtained from and propagated as recommended by ATCC. HSV-1 strains used in this study include wild-type KOS, n12 (67), and K5ΔZ (48). KOS stocks were prepared and titered on Vero cells. n12 stocks were grown in E5 cells, a Vero-based ICP4 complementing cell line (68). n12 stocks were titered on Vero and E5, as previously described (67). K5ΔZ stocks were grown and titered in G5 cells, a Vero-based VP5 complementing cell line, as described in reference 48. HD10.6 cells (69) were propagated and differentiated as described in reference 49. For differentiation, proliferating cells were initially seeded at a density of 2.5 × 10^4^ cells/cm^2^ on plastic petri dishes precoated overnight at 37° with 2% Matrigel (Corning), 1% poly-D-lysine (Gibco). Several coating compositions and procedures were explored, and this one produced the most stable monolayers. The day after seeding, proliferation media were removed, and differentiation media were added. The differentiation media were partially changed every 2 to 3 days. The differentiated cultures were used after 10–12 days of differentiation.

### Infection of MRC5 and HD10.6 cells

MRC5 cells were infected with the indicated virus at an MOI of 10 PFU/cell. Viral adsorption was done in 12.5 µL/cm^2^ in tricine buffered saline (TBS) for 1 h at room temperature. Viral inoculum was then removed, cells were washed with TBS twice, and 2% FBS media was added for the indicated incubation periods at 37°C. For HD10.6 cells, the adsorption volume was 60 μL/cm^2^ in neurobasal media (Gibco) plus glutaMAX (Gibco). After 1 h, the monolayers were washed 2× with neurobasal + glutamax. Differentiation media were added, and incubations were performed at 37°C. HD10.6 cells were infected with the indicated virus with 9.4 × 10^6^ PFU/cm^2^, unless otherwise specified.

### Quantification of viral DNA

To measure and calculate the average number of viral genomes per cell following infection, the media were removed, and nuclei were isolated by washing the cells in RSB-R (10 mM Tris HCl, pH 7.4, 10 mM NaCl, 3 mM MgCl_2_), suspending them in RSB-L (10 mM Tris HCl, pH 7.4, 10 mM NaCl, 3 mM MgCl_2_, 10% IGEPAL), and subjecting them to dounce homogenization. The generation of nuclei was monitored by staining with trypan blue and microscopy. The nuclei were pelleted by low-speed centrifugation, resuspended in TE (10 mM Tris-HCl, 1 mM EDTA, pH 8.0) containing 400 µg/mL proteinase K, 0.7% SDS, and incubated overnight at 65°C. Then, 0.4 mL TE was added, and the suspension was briefly sonicated in a bath sonicator to sheer the cellular DNA. The DNA was diluted 1:50 in nuclease-free water, and qPCR was performed to determine the number of viral and cellular genomes present using primers for viral genes, ICP8 and gC, and cellular gene, GAPDH (70). Standard curves were generated using CsCl gradient-purified KOS DNA and purified human male DNA. Samples and standards were run in triplicate.

### Immunofluorescence

A total of 2 × 10^5^ MRC5 cells were plated on glass coverslips in 35-mm dishes and infected with KOS. At 2 hpi, EdC was added to incubation media for 15, 30, 45, 60, and 90 min. Cells were fixed, click chemistry and immunofluorescent staining were performed, as previously described (38, 46). ICP4 antibody, the mouse monoclonal antibody 58S (71), was used to identify ICP4. Images were collected on an Olympus Fluoview FV1000 confocal microscope.

### ATAC-seq

We adapted and modified the procedure from Chen et al. (72). Two million MRC5 cells were plated in 60-mm dishes and infected as described above. At the indicated harvest times (post-adsorption), cells were crosslinked with 1% formaldehyde for 10 min followed by being quenched in 0.125 M glycine for 5 min. Cells were then washed with cold TBS and trypsinized for 5 min at 37°C. Cells were then scraped and spun at room temperature at 1,500 RPM for 5 min. Samples were resuspended in lysis buffer A (10 mM Tris-HCl, pH 7.4, 10 mM NaCl, 3 mM MgCl_2_) and spun at room temperature at 1,500 RPM for 5 min. Cells were resuspended in 1.8 mL lysis buffer A and 0.1% IGEPAL and dounced 10 times to isolate nuclei. An aliquot of 50 μL was taken to continue to ATAC-Seq, and the remaining volume is used to simultaneously quantify viral genomes using real-time quantitative PCR as described above. Aliquoted nuclei samples were then incubated on ice for 3 min with lysis buffer B (10 mM Tris-HCl, pH 7.4, 10 mM NaCl, 3 mM MgCl_2_, 0.1% IGEPAL, 0.1% Tween-20, 0.01% digitonin). Samples were spun at 4°C at 2,500 RPM for 5 min. Isolated nuclei were then resuspended in lysis buffer A with 0.1% Tween-20 and spun at 4°C at 2,500 RPM for 10 min. Nuclei were resuspended in 25 μL 2× buffer TD, 2.5 μL of TDE1 (Illumina #20034197), and 19 μL of ultra-pure nuclease-free water, and incubated at 37°C for 30 min while gently shaking. Following the tagmentation reaction, reverse crosslink solution (50 mM Tris-HCl, 1 mM EDTA, 1% SDS, 0.2 M NaCl, 5 ng/mL proteinase K) was added, and the suspension was incubated overnight at 65°C with gentle shaking. DNA was purified with a Monarch DNA and PCR CleanUp kit (NEB #TI030S). The purified DNA was then PCR amplified for 8–10 cycles. The individual amplified samples were quantified and quality checked using Agilent DNA 7500 kit and Bioanalyzer. Samples were multiplexed in equimolar concentrations and sequenced by the University of Pittsburgh Health Sciences Sequencing Core using Illumina NextSeq 2000. The sequencing runs were paired-end, 100 cycle, 4 × 10^8^ total reads.

### ATAC-seq analysis

Sequencing analysis was adapted from Dremel and DeLuca (21). Using Galaxy (73), fastq files were first aligned using bowtie2 to the mitochondrial genome to eliminate mitochondrial contamination. The remaining unmapped reads were then aligned to the human genome (hg38) (74) using bowtie2. This process was repeated to align the remaining reads to HSV-1 KOS strain (KT899744) (75). The resulting Bam files were used to generate bigwig files using deepTools bamcoverage with a bin size of 1. The bigwig files were either normalized to the number of viral reads to compare relative patterns or to the number of viral genomes per cell from the qPCR and the number of cellular reads to look at overall accessibility per genome. These bigwigs were then visualized using IGV. Internal replicates for each time point were compared using deepTools multiBigwigSummary to determine reproducibility. Bigwig files were averaged using deepTools bigwigCompare and compared with other time points using deepTools multiBigwigSummary to determine differences between wild-type and mutant viruses used at various time points. Viral and cellular alignments were also evaluated for insert size length in base pairs using Picard CollectInsertSizeMetrics. The quantity of insert size lengths per sample was summed, and each size length was divided by the total to determine the fraction of inserts at a given length. Samples for the same alignment and harvest time were averaged.

### RNA-Seq

RNA was isolated using the Ambion RNAqueous-4PCR kit. RNA sample concentration and quality were verified using the Agilent 6000 Nano kit. Libraries were generated using 1 µg RNA with the NEBNext Ultra II Directional Library Prep Kit (E7760S) and the Poly(A) mRNA Magnetic Isolation Module (E7490S).

### RNA-Seq analysis

Fastq files were uploaded and analyzed using the Galaxy web platform (73). Samples were first aligned to the human genome (hg38 canonical) using HISAT2 tool. Unaligned reads were then mapped to the HSV-1 strain KOS genome (KT899744) lacking repeating regions. The generated bam files mapped to the human genome and the viral genome, respectively, were then each analyzed for gene expression using featureCounts. Individual read counts per gene were then normalized mapped cell reads per kilobase of a given gene and sorted based on temporal class.

## Data Availability

All of the sequencing data used in this study are available at the Sequence Read Archive under the accession number PRJNA1387226.

## SUPPLEMENTAL MATERIAL

Supplemental material mbio.02987-25-s0001.pdf Figures S1-S4 and caption for Table S1.

Table S1 mbio.02987-25-s0002.xlsx RNA-seq values for Fig. 8.

ASM does not own the copyrights to Supplemental Material that may be linked to, or accessed through, an article. The authors have granted ASM a non-exclusive, world-wide license to publish the Supplemental Material files. Please contact the corresponding author directly for reuse.
