# Supplementary material for: The accessibility of the HSV genome during productive infection can vary in different cell types and affect the outcome of infection
Source: mBio. 2026 Feb 13;17(3):e02987-25. doi: 10.1128/mbio.02987-25 (PMC12977473; doi:10.1128/mbio.02987-25)
Supplement: Supplemental material — Figures S1-S4 and caption for Table S1. [file mbio.02987-25-s0001.pdf]

SUPPLEMENTAL INFORMATION

Nosek et al.

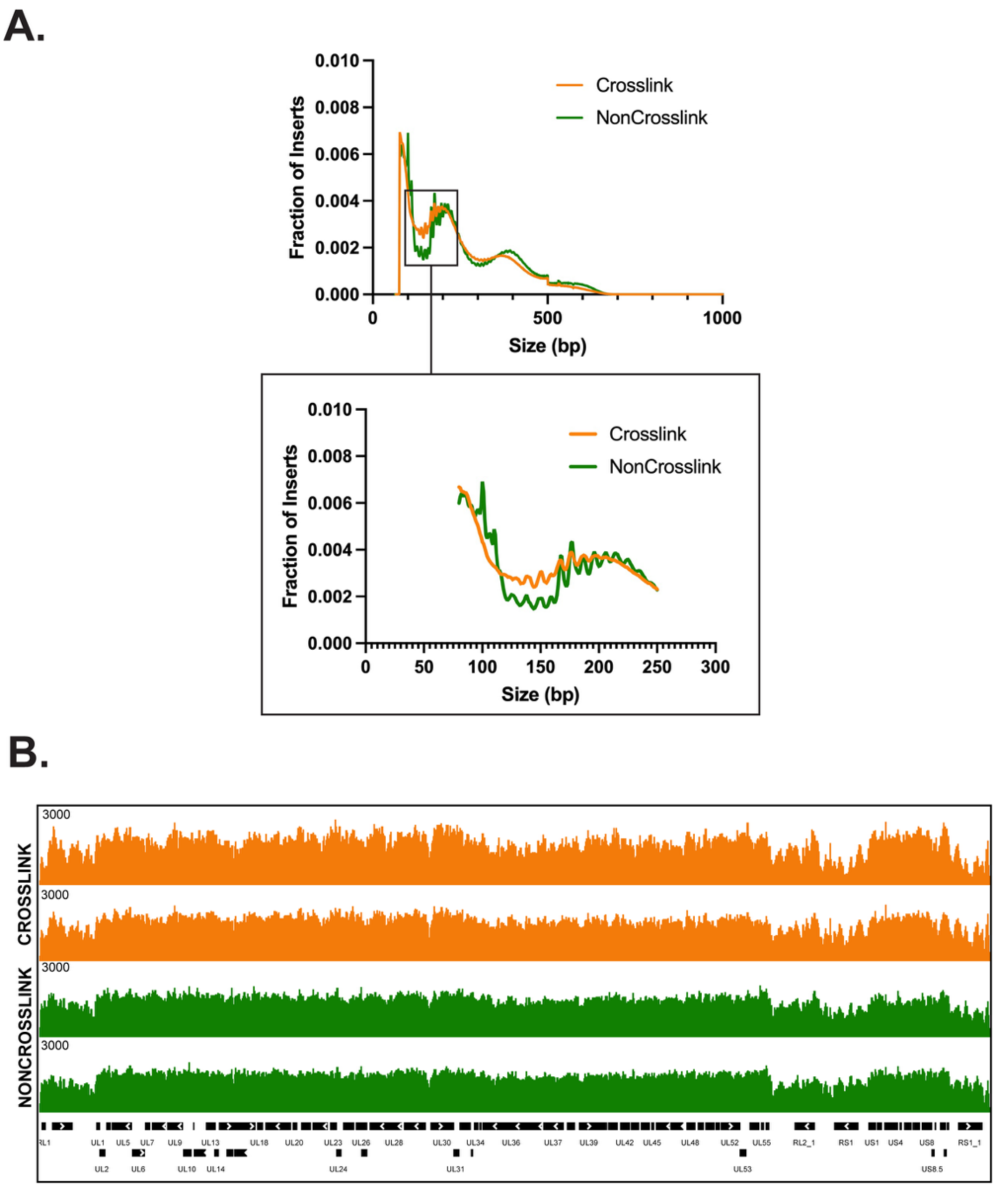

Supplemental Figure 1. Comparison of non-crosslinked and crosslinked ATAC-seq. A. Histogram plot of insert fragment size for crosslinked and non-crosslinked reads mapped to cellular genomes. The zoomed portion demonstrates the fine structure of the ATAC-seq data on the cellular genome. **B.** Alignment of viral reads to the viral genome. Bigwigs were normalized to sequencing depth and viral reads.

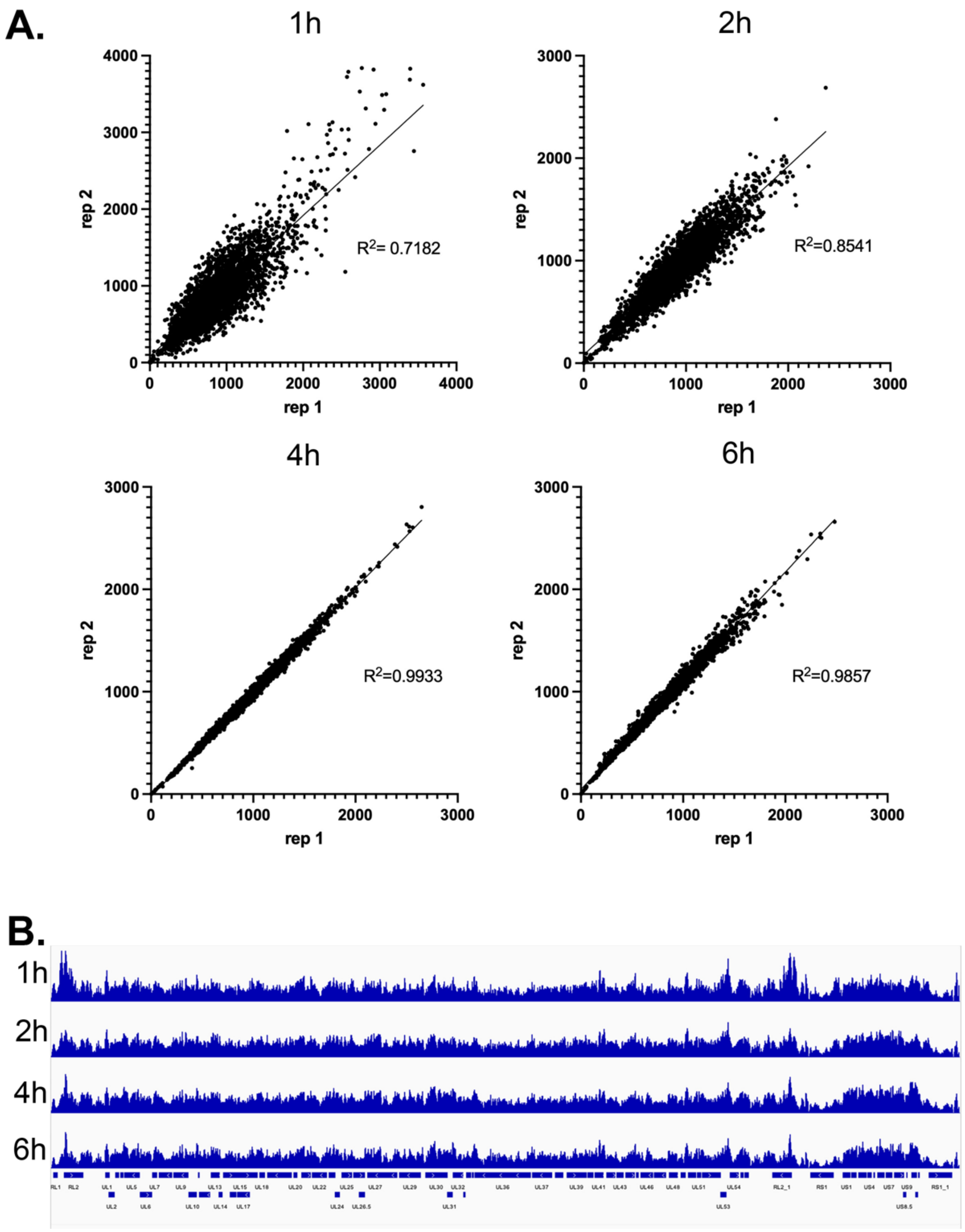

Supplemental Figure 2. Comparison of genome accessibility before and after the onset genome replication. A. Reproducibility plots for the duplicate samples for each of the time points. **B.** ATAC-Seq bigwig alignment to the KOS viral genome normalized to viral read. The maximum for all four plots is 4,000 reads.

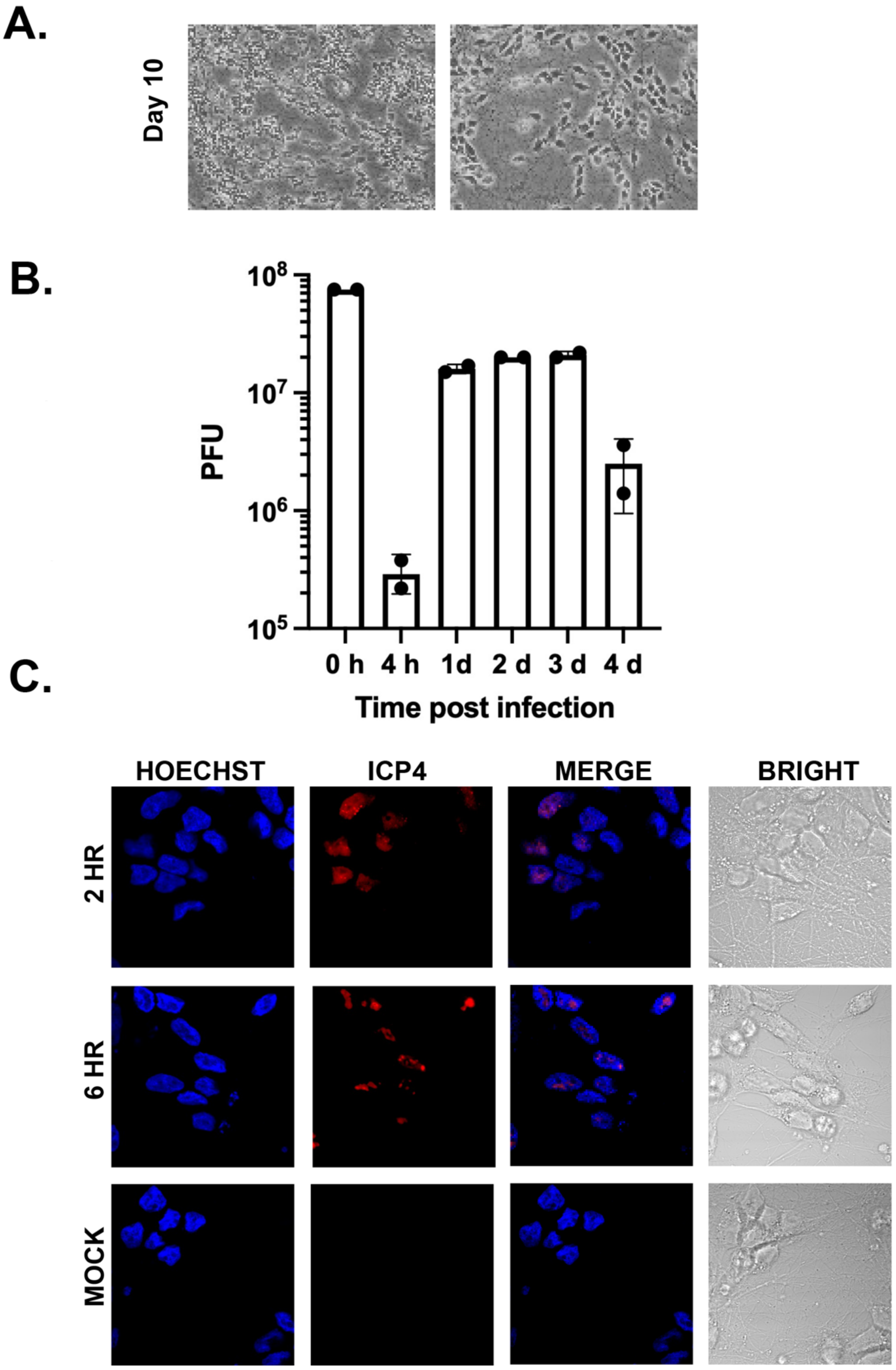

Supplemental Figure 3. Parameters of infection in differentiated HD10.6 cells. A. Bright field images demonstrating the morphology of HD10.6 cells differentiated for 10

days. Images were taken at a magnification of 200x and 500x. B. 35 mm dishes differentiated HD10.6 cells were infected with 2.5 x107 virus (KOS) and harvested at the indicated times. Virus yield was determined by plaque assay on Vero cells. C. Differentiated HD10.6 on coated glass coverslips in 35 mm dishes were infected with 2.5 x10^7 virus (KOS) and fixed at 2- and 6-hours post infection. ICP4 was visualized with immunofluorescence in comparison with the cellular DNA visualized using Hoechst. Bright field images of infected cells were taken concurrently to capture infected cell morphology at these times. Mock cells were treated to all the same conditions.

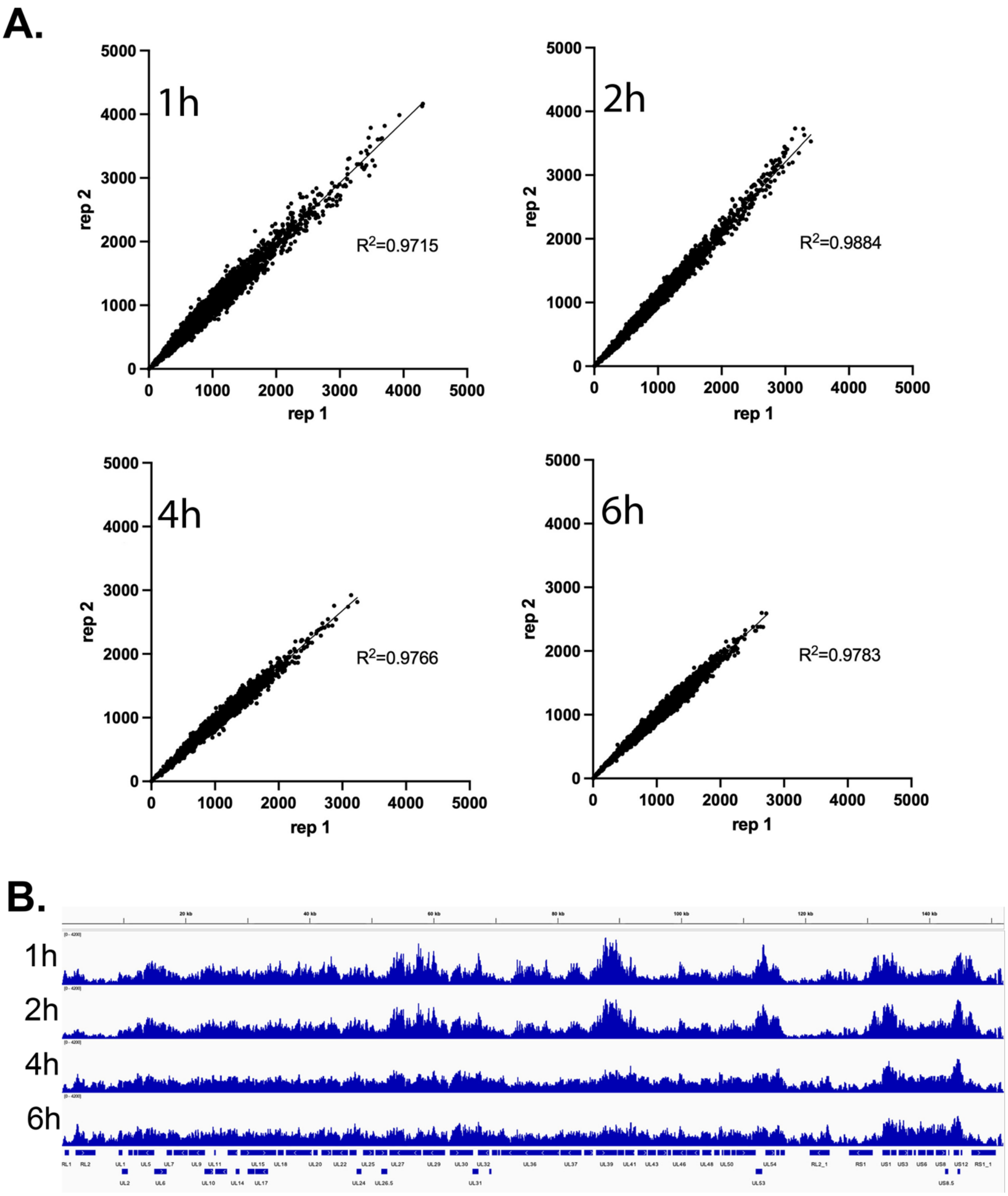

Supplemental Figure 4. Viral genome accessibility in differentiated HD10.6 cells.

A. Reproducibility plots for the duplicate samples for each of the time points. **B.** ATAC-Seq bigwig alignment to the KOS viral genome normalized to viral reads.

Supplemental Table 1. RNA-seq values for Fig. 8
